# Genome Sequence of *Zymomonas mobilis* subsp. *mobilis* NRRL B-1960

**DOI:** 10.1128/genomeA.00562-17

**Published:** 2017-07-27

**Authors:** Katherine Chacon-Vargas, Alexandra A. Chirino, Meghan M. Davis, Sophia A. Debler, Wynn Raphael Haimer, Justin J. Wilbur, Xiaoli Mo, Baxter W. Worthing, Ethan G. Wainblat, Shu Zhao, John G. Gibbons

**Affiliations:** Biology Department, Clark University, Worcester, Massachusetts, USA

## Abstract

*Zymomonas mobilis* subsp. *mobilis* is an efficient ethanol producer with application for industrial production of biofuel. To supplement existing *Z. mobilis* genomic resources and to facilitate genomic research, we used Oxford Nanopore and Illumina sequencing to assemble the complete genome of the beer spoilage isolate *Z. mobilis* subsp. *mobilis* strain NRRL B-1960.

## GENOME ANNOUNCEMENT

*Zymomonas mobilis* subsp. *mobilis* is a Gram-negative alphaproteobacterium capable of highly efficient ethanol production through the Entner-Doudroff pathway (1). *Z. mobilis* has garnered interest for its prospective biofuel application, and it is utilized in industrial-scale fermentations to produce bionic acid, levan, and sorbitol (2, 3). The genomes of several natural isolates of *Z. mobilis* from North America, South America, Europe, and Africa have been previously sequenced (4–8). Here, we sequenced the genome of *Zymomonas mobilis* subsp. *mobilis* NRRL B-1960 (NCIMB 8227), originally isolated from “bad beer” in the United Kingdom (1), to facilitate population and comparative genomics research of this industrially important species.

*Z. mobilis* NRRL B-1960 was grown in 0.5% yeast extract and 2% glucose liquid medium at 30°C for 24 h. DNA was extracted using the PureLink microbiome DNA purification kit (Thermo Fisher Scientific), and DNA integrity was evaluated via gel electrophoresis. We constructed an Oxford Nanopore (ONT) rapid 1D library from 500 ng of DNA, according to the manufacturer’s instructions (kit version RAD002) and performed sequencing on two flow cells (R9 version) of the MinION device. Poretools version 0.6.0 (9) was used to convert ONT data from fast5 to fastq format. We performed error correction of ONT reads using Canu version 1.4 (10) and generated a total of 12,302 corrected reads, with an average read length of 4,152 bp, representing ~25× coverage. A paired-end 101-bp Illumina library was constructed and sequenced at Macrogen (Rockville, MD) from 1 µg of DNA. Illumina sequence data were deduplicated using Tally (11), adapter and quality trimmed using Trim Galore version 0.4.3 (https://www.bioinformatics.babraham.ac.uk/projects/trim_galore/), and errors corrected using SPAdes version 3.10.0 (12), resulting in 14,838,803 paired-end reads, representing ~1,400× coverage. We incorporated both sequence data types in SPAdes to produce an assembly using k-mer sizes of 27, 37, 47, 57, 67, 77, 87, and 97 (12). Last, preassembly-improved Illumina data was used for genome polishing via Pilon version 1.21 (13).

We assembled the genome into a single scaffold consisting of 2,045,798 bp, with a GC content of 46.09%. Two additional scaffolds were assembled which correspond to plasmid sequences. The larger plasmid sequence, pZMO1960_1, is 34,463 bp, with a GC content of 45.77%, and has an ~5.5-kb region nearly identical to a *Z. mobilis* CP4 plasmid (GenBank accession no. CP006894) (6). The other plasmid sequence, pZMO1960_1A, is 1,743 bp, with a GC content of 38.15%, and it is identical to the *Z. mobilis* NCIMB 11163 plasmid pZMO1A (GenBank accession no. GQ293074) (5). The RAST server (14, 15) was used for genome annotation, and clustered regularly interspaced short palindromic repeat (CRISPR) arrays were predicted using CRISPRFinder (16). The genome contains 1,971 protein-coding genes, 3 rRNA gene loci, 51 tRNA-encoding genes, and 3 CRISPR arrays.

Phylogenetic analysis of 74,852 polymorphic sites across the sequenced natural isolates of *Z. mobilis* indicates that the NRRL B-1960 genome is most closely related to the NCIMB 11163 strain, which was isolated from spoiled beer in England (5). We conservatively identified 11 genes unique to the NRRL B-1960 genome, including 2 TonB-dependent receptor-encoding genes, via BLAST (17) searches of the NRRL B-1960 genes against the 5 sequenced *Z. mobilis* genomes (4–8).

### Accession number(s).

The *Zymomonas mobilis* subsp. *mobilis* NRRL B-1960 genome was assigned GenBank accession number CP021053 for the chromosome and accession numbers CP021791 and CP021792 for the plasmids.
